# Supplementary figures and images for: Whole-genome single-nucleotide polymorphism (SNP) marker discovery and association analysis with the eicosapentaenoic acid (EPA) and docosahexaenoic acid (DHA) content in Larimichthys crocea
Source: PeerJ. 2016 Dec 21;4:e2664. doi: 10.7717/peerj.2664 (PMC5180582; doi:10.7717/peerj.2664)

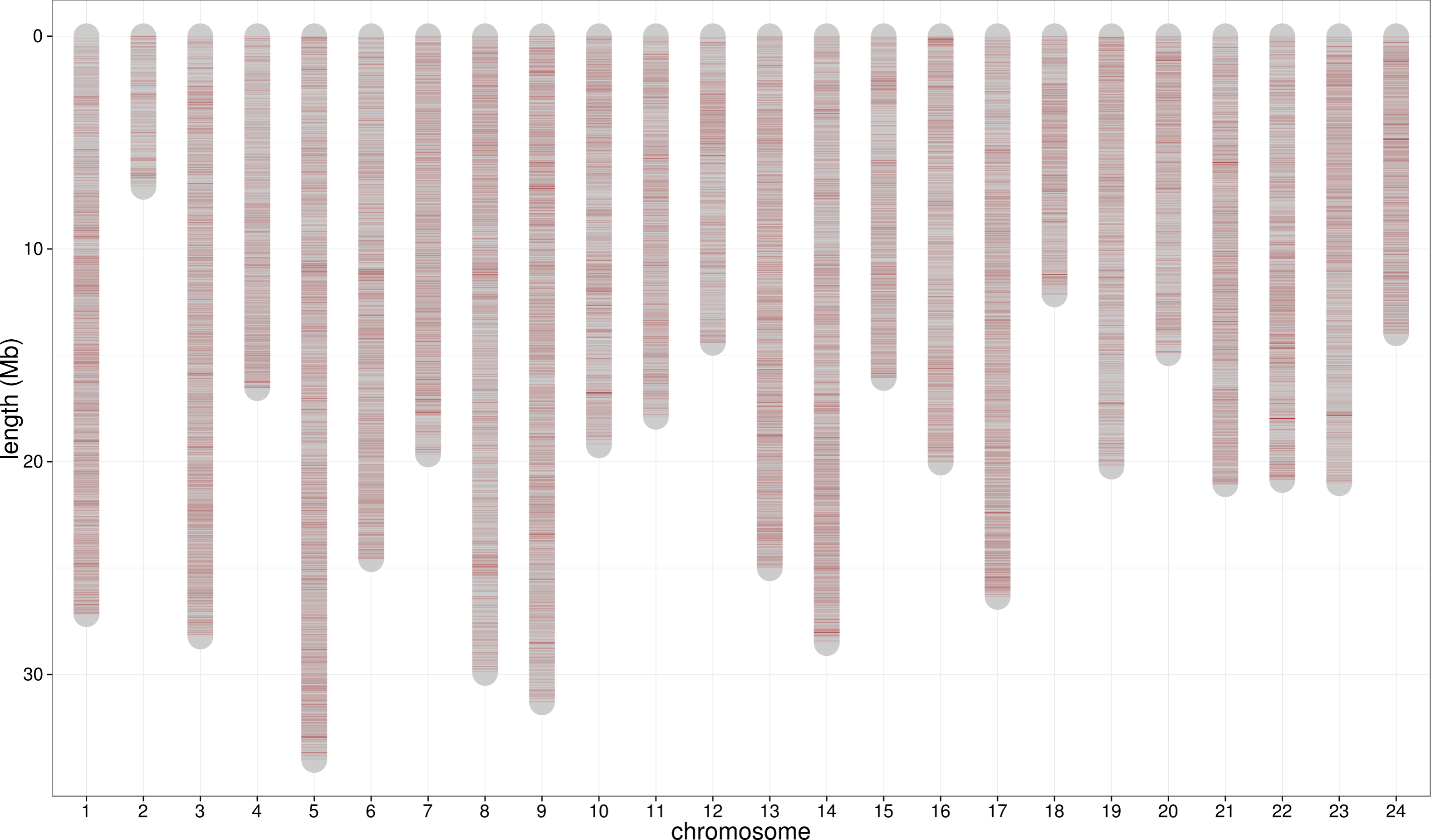

Supplement: Figure S1 [file peerj-04-2664-s005.pdf]

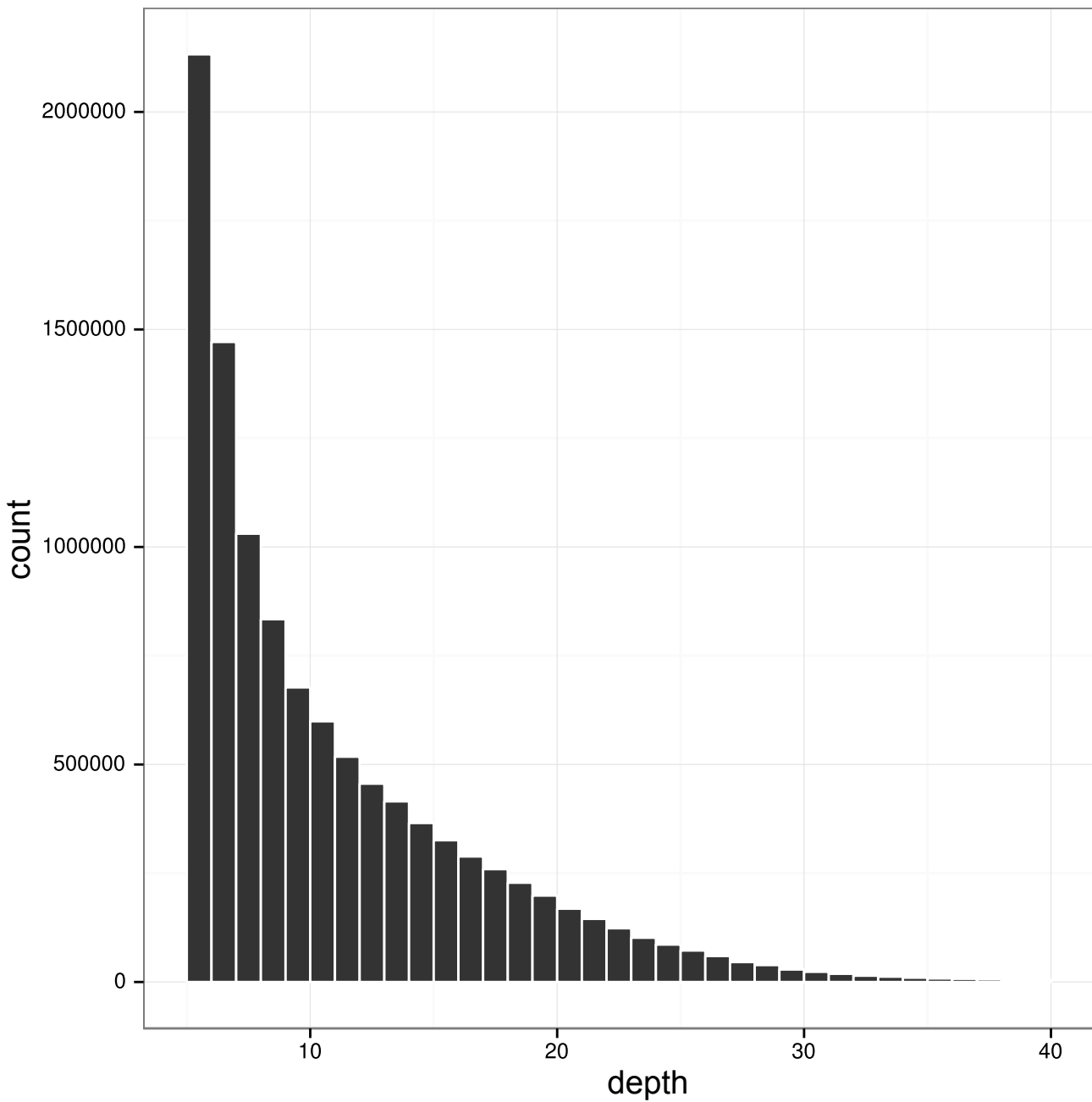

Supplement: Figure S2 [file peerj-04-2664-s006.pdf]

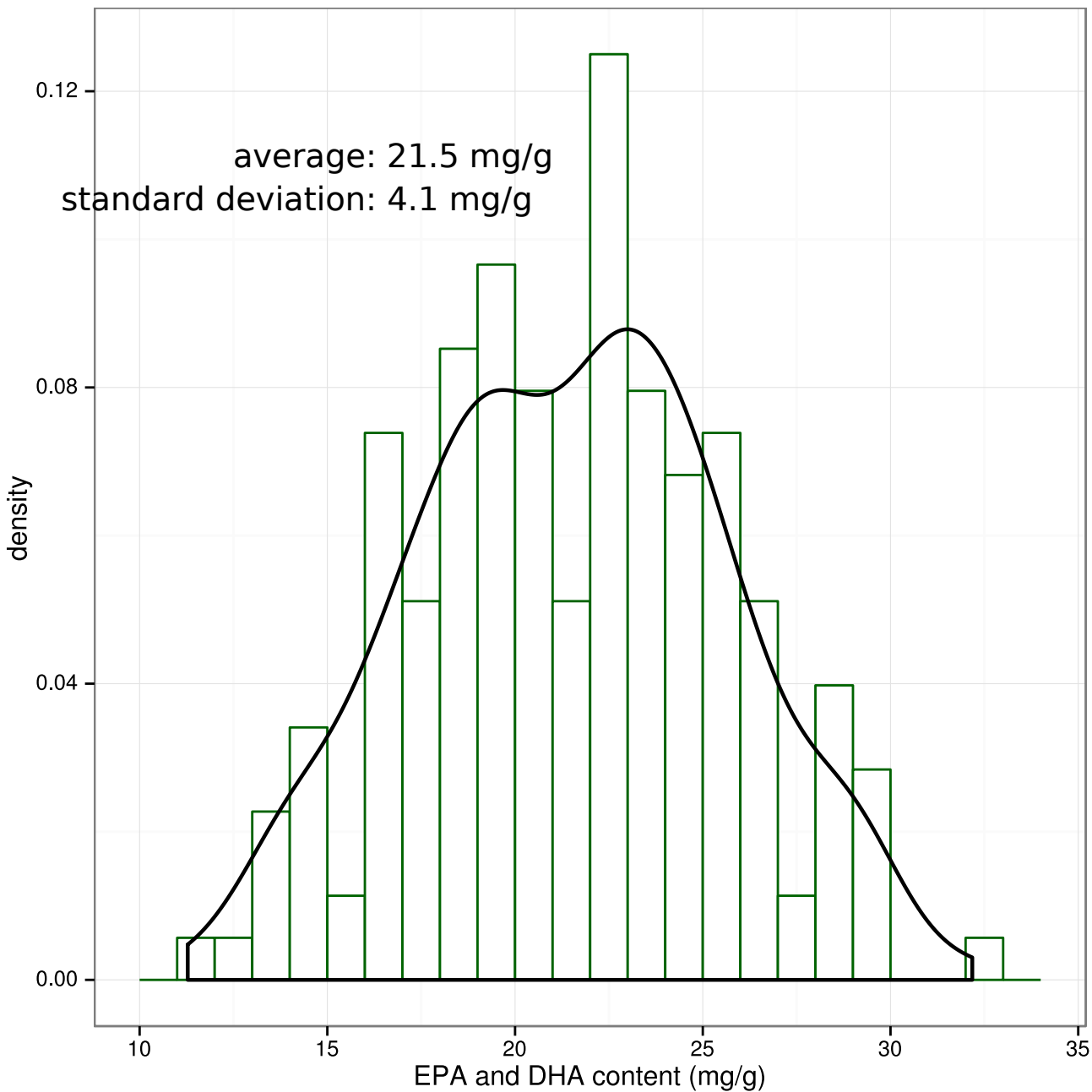

Supplement: Figure S3 [file peerj-04-2664-s007.pdf]

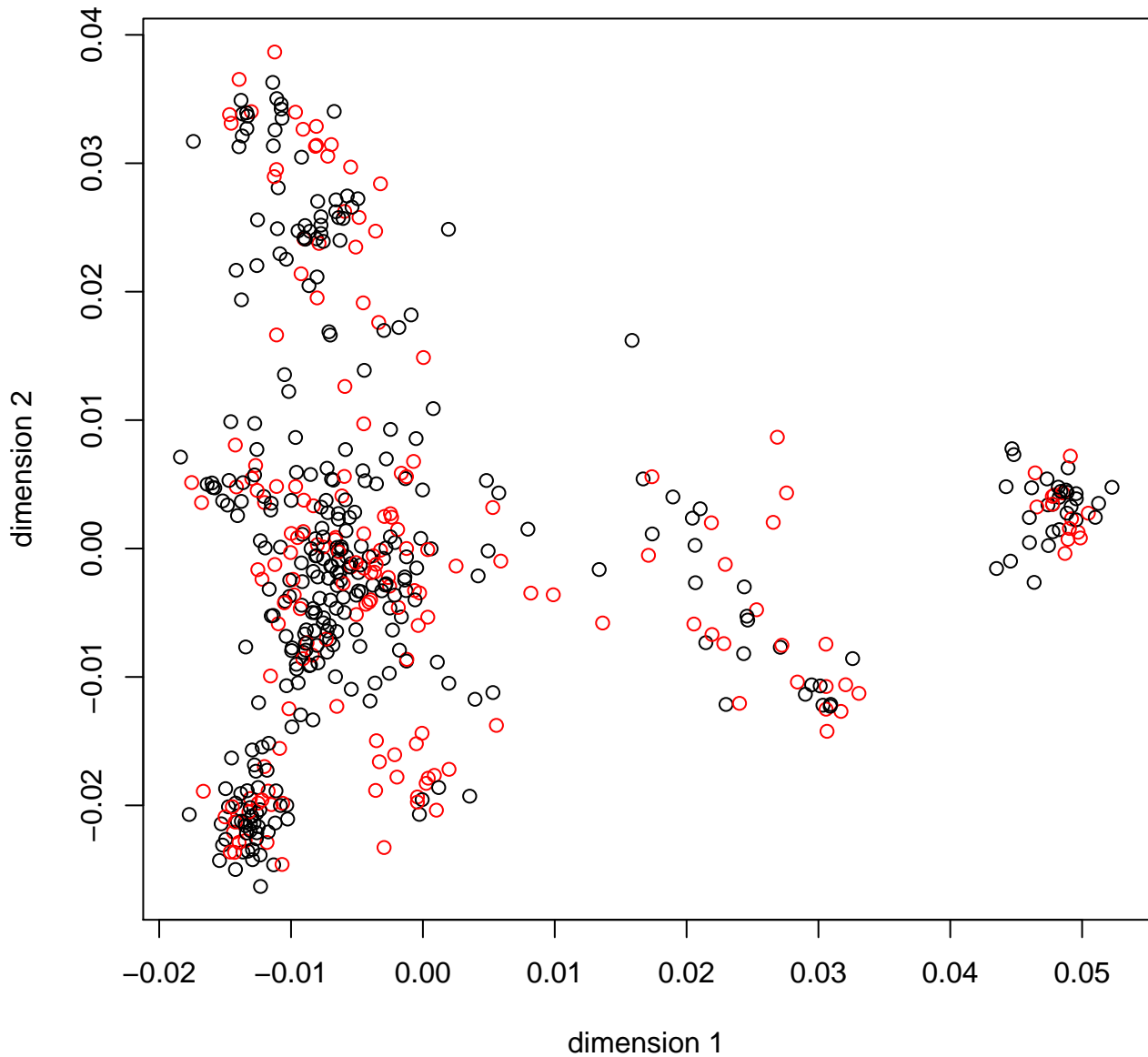

Supplement: Figure S4 [file peerj-04-2664-s008.pdf]
